# Partial Extirpation of the Parotid Gland

**Published:** 1878-03

**Authors:** F. W. Epley

**Affiliations:** New Richmond, Wis.


*Partial Extirpation of the Parotid Gland.*

Miss S.——, a school teacher, had suffered for some time from a tumor under the left ear, which was painful at times, and had a decidedly malignant "feel;" had been steadily, though slowly, increasing in size until May 1st, 1877, when it came under the observation of Drs. Hoyt and King, of Hudson. Upon May 15th it was decided to operate for the removal of the diseased growth, which proved to be nearly the whole of the parotid gland. Patient being anæsthetized and a "T" shaped incision made over the tumor, one incision from the "lobule" downwards for about two inches, and one horizontally from mastoid process of temporal bone to one and one-half inches in front of the ear, its lower border and the flaps were dissected up, when the tumor was exposed. Then manipulating very carefully with the handle of the scalpel the diseased tissues were separated from the adjoining structures to which they were quite intimately adherent, until the vagus nerve, external carotid artery, and external jugular vein were plainly exposed to view, great care being exercised not to destroy any more of the filament of the facial nerve than was absolutely unavoidable. The tumor being thus carefully loosened from its bed a ligature was thrown around its pedicle and its connections severed. The hemorrhage was inconsiderable after having ligated a small branch from the facial artery. The wound was closed by four or five stitches, healing kindly in about ten days, except a small fistulous opening which discharged the natural secretion of the gland remaining. This was cauterized several times with nitrate of silver, and finally its edges were scarified and strapped closely together with adhesive plaster when it closed entirely, and up to the present date (Feb. 15, '78) no return of the disease has shown itself. There was only very slight paralysis of the facial muscles. F. W. EPLEY, M. D.

NEW RICHMOND, WIS.

